# Potential Health Benefits of Olive Oil and Plant Polyphenols

**DOI:** 10.3390/ijms19030686

**Published:** 2018-02-28

**Authors:** Monika Gorzynik-Debicka, Paulina Przychodzen, Francesco Cappello, Alicja Kuban-Jankowska, Antonella Marino Gammazza, Narcyz Knap, Michal Wozniak, Magdalena Gorska-Ponikowska

**Affiliations:** 1 Department of Medical Chemistry, Medical University of Gdansk, 80-211 Gdańsk, Poland; gorzynikdebicka@gmail.com (M.G.-D.); p.e.przychodzen@gumed.edu.pl (P.P.); alicjakuban@gumed.edu.pl (A.K.-J.); narcyz@gumed.edu.pl (N.K.); mwozniak@gumed.edu.pl (M.W.); 2 Department of Experimental Biomedicine and Clinical Neurosciences (BioNeC), University of Palermo, 90127 Palermo, Italy; francapp@hotmail.com (F.C.); antonella.marino@hotmail.it (A.M.G.); 3 Euro-Mediterranean Institute of Science and Technology (IEMEST), 90136 Palermo, Italy; 4 Institute of Biomaterials and Biomolecular Systems, Department of Biophysics, University of Stuttgart, 70569 Stuttgart, Germany

**Keywords:** olive oil, *Olea europea*, polyphenols, oleuropein, hydroxytyrosol, anticancer therapy

## Abstract

Beneficial effects of natural plant polyphenols on the human body have been evaluated in a number of scientific research projects. Bioactive polyphenols are natural compounds of various chemical structures. Their sources are mostly fruits, vegetables, nuts and seeds, roots, bark, leaves of different plants, herbs, whole grain products, processed foods (dark chocolate), as well as tea, coffee, and red wine. Polyphenols are believed to reduce morbidity and/or slow down the development of cardiovascular and neurodegenerative diseases as well as cancer. Biological activity of polyphenols is strongly related to their antioxidant properties. They tend to reduce the pool of reactive oxygen species as well as to neutralize potentially carcinogenic metabolites. A broad spectrum of health-promoting properties of plant polyphenols comprises antioxidant, anti-inflammatory, anti-allergic, anti-atherogenic, anti-thrombotic, and anti-mutagenic effects. Scientific studies present the ability of polyphenols to modulate the human immune system by affecting the proliferation of white blood cells, and also the production of cytokines or other factors that participate in the immunological defense. The aim of the review is to focus on polyphenols of olive oil in context of their biological activities.

## 1. Beneficial Effects of Polyphenols

As the name suggests, polyphenols are natural, synthetic, or semisynthetic organic compounds with multiple phenolic groups in the structure. It means that polyphenols typically contain one or more aromatic rings with hydroxyl groups attached to them [1,2]. There is a growing body of evidence for beneficial roles of natural plant polyphenols in the human body. Natural bioactive polyphenols are compounds of varied chemical structures. Polyphenols are arguably the largest group of chemical substances in the plant kingdom. There are more than 8000 different polyphenolic structures known, including several hundred isolated from edible plants [3,4]. Their sources are, among others, fruits, vegetables, nuts and seeds, roots, bark, leaves of different plants, herbs, whole grain products, processed foods, as well as tea, coffee, and red wine. These compounds are characterized by a broad spectrum of biological activities. The beneficial impact of vegetables, fruits, and herbs on human health has been well known for centuries. Today, we understand the reasons for that as many plant-derived products are rich in nutrients, vitamins, minerals, and very importantly, bioactive polyphenols. Some vitamins as well as polyphenols present powerful antioxidant and anti-inflammatory properties that make them natural and efficient anticancer agents to be found in a well-balanced diet. Unlike vitamins and minerals, polyphenols are not the essential elements of the primary plant metabolism. Natural polyphenolic compounds are rather products of the secondary plant metabolism. Anyhow, they do play critical metabolic roles in the human organism [2,5,6,7].

Polyphenols were determined to reduce morbidity and/or slow down the progression of cardiovascular, neurodegenerative, and cancer diseases. The mechanism of action of polyphenols strongly relates to their antioxidant activity. Polyphenols are known to decrease the level of reactive oxygen species in the human body. Other than that, health-promoting properties of plant polyphenols comprise anti-inflammatory, anti-allergic, anti-atherogenic, anti-thrombotic, and anti-mutagenic effects [8]. There is a body of research presenting their ability to modulate the human immune system by affecting the proliferation and activity of white blood cells, as well as the production of cytokines or other factors that participate in the immunological defense [9].

A daily intake of polyphenols ranges from 0.1 to 1.0 g per day [10] with the main dietary source being fruits and vegetables, as well as herbs, spices, seasonings, coffee, tea, or wine [11]. The polyphenols of olive oil are especially interesting with respect to their well-established beneficial effects on human health and metabolism, as well as the popularity of olive oil in many different diets, and specifically the Mediterranean cuisine. Herein, we mostly focus on the anticancer properties of polyphenols available from olive oil.

## 2. Chemical Composition of Olive Oil

**Figure 1:**
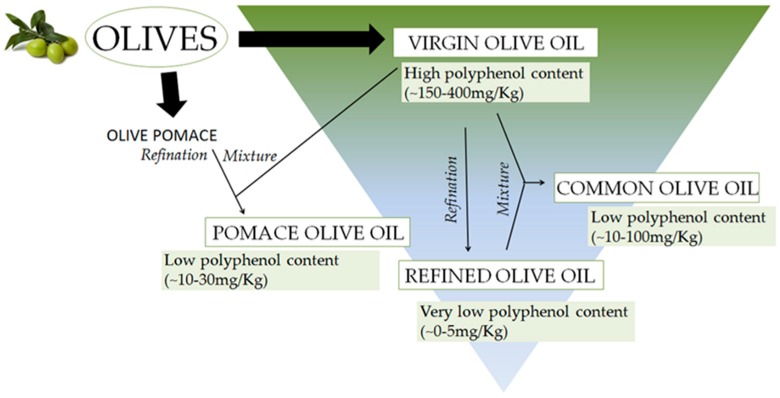
The concentration of polyphenols in differnet kinds of oilve oil depending on technological process of the oil extraction [25].

The chemical composition of olive oil varies depending on the extraction technology that is applied in order to obtain oil form the fruits (Figure 1). The process of extraction of olive oil depends on crushing olives and then separating the oil from the fruit pulp under elevated pressure. Additionally, olive oil can be extruded, post-pressured, re-pressed with or without the use of hot water. The olive oil obtained from this kind of process is usually characterized by stronger color intensity, weaker aroma, and a higher content of free fatty acids [12,13,14].

The oil obtained by chemical extraction can be used for consumption only after refining. A refining process is meant to purify the extracted oil from any residual solvent and other impurities. Refined olive oil is devoid of vitamins, polyphenols, phytosterols, and other low molecular natural ingredients [15]. Extra virgin olive oil by its low yield is more expensive than other types of olive oil, but it contains the highest level of polyphenols [16]. Due to the removal of free fatty acids, extra virgin olive oil has a delicate flavor, aroma, and light color [14,17,18,19,20,21,22,23,24]. Another interesting feature affecting virgin olive oil properties is filtration. Unfiltered olive oil preserves additional polyphenols of higher polarity that are typically lost with small amounts of water that are removed upon filtration.

Due to multiple technological processes, the content of polyphenols may vary in olive oil. Figure 1 represents the level of polyphenols in olive oil dependent on the technological process of olive oil production.

Olive oil mostly consists of triacylglycerols (98–99%). Triacylglycerols (TGA) are a diverse group of glycerol esters with different fatty acids. The predominant fatty acid present in olive oil TGAs is monounsaturated oleic acid (up to 83% *w*/*w*). There is also palmitic acid, linoleic acid, stearic acid, and palmitoleic acid making up the remainder of olive oil TGAs. There is a plethora of lipophilic or amphiphilic microconstituents present in virgin olive oil, among them, phytosterols, squalene, tocopherols, phenolic compounds, terpenic acid derivatives, etc. [26,27,28]. Phenolic compounds occur in the form of: phenolic acids or alcohols, oleuropein derivatives, lignans, and flavonoids. In olive oil, the content of polyphenols ranges from 50 to 1000 mg/kg. As a matter of fact, it depends on the agronomic factors, the ripeness of olives, as well as extraction technology, along with storage or packaging processes [29,30,31,32,33].

The flesh of healthy olives contains about 2–3% of phenolic substances in the form of glucosides and esters. Virgin olive oil contains about 500 mg/L of polyphenols. The quantity and quality of polyphenols in olive oil is closely related to the process of olive milling and further processing. Therefore, virgin olive oils have substantially higher amounts of polyphenols than refined olive oils [30,33]. The phenolic compounds in olive oil are mostly glycides (e.g., oleuropein), alcohols and phenols (tyrosol, hydroxytyrosol), and also flavonoids [26,28,34]. Phenolic compounds are mainly responsible for the characteristic gustatory property of virgin olive oil, namely the bitter taste. Some micro constituents of olive oil are soluble in water, and thus, the content of phenolic compounds that are present in olive oil depends to a large extent on the extraction process [29,34,35,36].

## 3. Beneficial Effects of Olive Oil and Olive Leaf Extract

Conventional medicine and phytotherapy both use olive leaf extracts to treat and prevent arterial hypertension or as diuretics and antiseptics [37,38]. Many studies on olive leaf extract showed that it is able to lower the blood pressure in animals as well as to increase blood flow through the coronary arteries, slow down the heart rate and normalize intestinal muscle contractions [37,39,40]. In clinical studies including patients with first-degree hypertension treated with olive leaf extract, the tendency to lowering the blood pressure was observed. Twice-daily dosing of 500 mg (1 g/day) of the olive leaf extract was as effective as an alternative medicine that is typically prescribed in order to lower blood pressure [41].

Recently, a chemopreventive activity of olive oil has been attributed to its unique phenolic compounds represented by phenolic alcohols like hydroxytyrosol (3,4-dihydroxyphenylethanol: 3,4-DHPEA) and tyrosol (*p*-hydroxyphenylethanol: p-HPEA), and their secoiridoid derivatives 3,4-DHPEA-EA (oleuropein aglycon), *p*-HPEA-EA (ligstroside aglycon), 3,4-DHPEA-EDA, *p*-HPEA-EDA (oleocanthal), and oleuropein [42].

Anticancer properties of olive oil seem to correlate with the antioxidant activity of phenolic and polyphenolic compounds present therein that are capable of scavenging free radicals and reactive oxygen species. Oleuropein, tyrosol, hydroxytyrosol, verboscoside, ligustroide, demethyleuropein were all proven to protect against the coronary artery disease [43,44,45,46] or cancer [43,47,48]. They also display antimicrobial and antiviral effects [43,49,50,51]. Antioxidant and anti-atherogenic effects of olive oil polyphenols, like oleuropein and hydroxytyrosol, have been vastly confirmed in the literature [52,53].

### 3.1. Health Benefits of Hydroxytyrosol

Hydroxytyrosol (HT) belongs to polyphenols, which are abundant in olives (*Olea europea* L.) and consequently in virgin olive oil. Its beneficial properties for human health are strongly related to the ability of the molecule to scavenge free radicals and reactive oxygen/nitrogen species as well as to activate endogenous antioxidant systems in the body. Free radical scavenging properties of HT have been convincingly confirmed in studies on rats with alloxan-induced diabetes mellitus [54].

The studies on the 3T3-L1 adipocyte cell line have shown that HT stimulates mitochondrial biosynthesis which is reduced in the course of diabetes mellitus. Most likely, HT increases mitochondrial biosynthesis pathway via upregulation of PGC-1α. Relatively low concentrations of hydroxytyrosol in adipocytes increase the expression of all mitochondrial respiratory chain complexes, including ATP synthase. HT protects mitochondria against the reduction of mitochondrial DNA synthesis, and modulates activity of the critical transcription factors, such as Nrf1 (nuclear respiratory factor 1) and Tfam (transcription factor A, mitochondrial). All of these unique properties of HT are attributed to a potential risk reduction for developing type 2 diabetes mellitus [55].

### 3.2. Beneficial Properties of Oleuropein

Oleuropein belongs to a group of coumarin derivative, secoiridoids [56]. It was found to be effective against various strains of bacteria, viruses, fungi and also molds or even parasites. Moreover, it inhibits platelet aggregation [57]. It is also a major constituent of a patented formulation of an endothelial proliferation inhibitor. Oral treatment with oleuropein results in a decreased number of blood vessels proving strong anti-angiogenic properties [58]. Phenolic compounds (oleuropein, protocatechuic acid) of virgin olive oil have also been shown to inhibit macrophage-mediated LDL oxidation [59]. Leaf and olive fruit extracts containing oleuropein protect insulin-producing β-cell line (INS-1) against the deleterious effect of cytokines [60].

## 4. Antineoplastic Properties of Olive Oil Polyphenols and the Mechanism of Action

**Figure 2:**
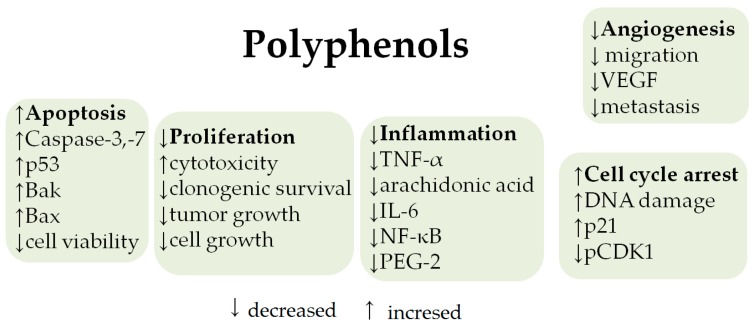
Anticancer mechanisms of polyphenols from olive oil [62].

Research has shown that some natural plant-derived polyphenols can directly or indirectly prevent cells from the initiation of neoplastic transformation due to xenobiotics and carcinogenic factors, and thus contribute to a lower risk of developing cancer. Carcinogenesis is characterized by a change in the transcriptional activity of many genes, and consequently in the biological function of the proteins that are encoded by those genes. There are many studies pointing out an essential role of polyphenolic compounds as derived from vegetables, fruits, or herbs in the regulation of epigenetic modifications, resulting in the antiproliferative protection [61]. Mechanisms of anticancer activity of olive oil polyphenols are presented in Figure 2.

Researchers reported anticancer properties of olive leaf extract in an animal skin cancer model. The extract caused cancer cell death, starting with early apoptosis and completing by the following necrosis [63,64]. Polyphenols from olive leaf extract showed synergistic effects as combined with standard chemotherapeutic agents [63].

Our studies on polyphenols of olive oil (especially oleuropein and hydroxytyrosol) also confirmed their anticancer potential on proliferation and cell death of number of cancers (*osteosarcoma, neuroblastoma*, breast cancer). The obtained data indicate as well the plausible synergism between polyphenols of olive oil and standard chemotherapeutics (data not published yet).

Epidemiological studies show that people of the Mediterranean region have a lower incidence of several cancers when compared to other populations [65]. The consumption of olive oil is an important factor in the Mediterranean diet, and is generally believed to be beneficial for health [66]. Olive oil consumption was proven to prevent from colorectal cancer, breast cancer and skin cancer [67,68,69]. Several mechanisms of antitumor activity of virgin olive oil have been determined. Interestingly, olive oil polyphenols were proven to protect biological membranes against oxidative modification and losing structural integrity. Similarly, polyphenolic compounds decreased oxidative damage to cellular DNA effectively decreasing promotion of colon cancer. In addition to that, HT inhibited lipooxygenase, the enzyme responsible for leukotriene synthesis, and thus modulating the inflammatory response. An alteration of the hormonal status was also observed, specifically due to antiestrogenic effects of lignans that are present in virgin olive oil [70,71].

There are pleiotropic effects of olive oil polyphenols, as observed on the molecular level. It seems however that the antioxidant potential of olive oil is the primary factor contributing to protection against cancer [72,73].

Olive oil can also protect against carcinogenic agents that are abundant in the environment and food. Again, the most important protection mechanism is alleviation of oxidative stress phenomena. It is worth mentioning that oleic acid being the major fatty acid present in olive oil TAGs is much less susceptible to oxidation than polyunsaturated acids that are abundant in seed oils. High content of antioxidant polyphenols (hydroxytyrosol, oleuropein) makes virgin olive oil relatively stable and resistant to oxidation, and thus minimizing the risk of formation of potentially carcinogenic products of lipid peroxidation upon storage. Moreover, olive oil polyphenols have been reported to induce beneficial epigenetic modifications [74] and miRNA expression pattern, lowering cancer risk [75,76].

Studies have shown that both oleuropein and hydroxytyrosol, inhibited angiogenesis and specifically endothelial tube formation by HUVEC cells on Matrigel as well as lowered migration in wound healing assays [77].

### 4.1. Anticancer Activity of Hydroxytyrosol

Treatment of human colon adenocarcinoma cells with olive oil polyphenols significantly inhibited cell proliferation [78,79,80]. In spite of the relatively low concentrations of hydroxytyrosol in olive oil, polyphenols are in high micromolar concentration range in the colon after gastric hydrolysis and colonic fermentation of secoiridoids that are naturally present in olive oil [81]. In colon cancer cells HT reduces epidermal growth factor receptor (EGFR) level by promoting its degradation. EGRF is one of the key receptors triggering colon carcinogenesis as it regulates the proliferation, apoptosis, angiogenesis, and invasion of cancer cells [8,82]. Moreover, HT was found to be an effective cytotoxic agent in breast cancer cell models. It inhibited cell cycle in the G0/G1 phase by decreasing the level of cyclin D1 [8,62].

### 4.2. Oleuropein as Anticancer Agent

There are numerous studies confirming the anticancer activity of oleuropein, as observed in human cancer cell lines, such as: breast adenocarcinoma (cell line MCF-7, MDA) [83,84,85,86,87], melanoma (cell line RPMI 7951) [86], urinary bladder carcinoma (cell line T-24) [86], colorectal adenocarcinoma (cell line HT 29, Caco-2, LoVo) [86,88,89], prostate cancer (cell line TF1) [87], lung carcinoma (cell line A549) [90], glioblastoma (cell line LN 18), renal cell adenocarcinoma (cell line 786-O) [87], and glioma (cell line U251, A172) [91].

Oleuropein was found to display antiproliferative, proapoptotic effects [92], overall anticancer activity [93], and specifically an ability to decrease cancer cell viability [94], as well as induction of cancer cell apoptosis [95,96,97,98]. Oleuropein induces apoptosis in breast cancer cells (MCF-7) via the *p53*-dependent pathway and through the regulation of *Bax* and *Bcl2* genes. Therefore, oleuropein may have a great therapeutic potential for breast cancer treatment [99]. Interestingly, oleuropein has been reported to inhibit aromatase, a cytochrome P450 enzyme, which is an important pharmacological target in breast cancer therapy [100]. Moreover, it increases cancer cell sensitivity to trastuzumab (>1000-fold increase) in SKBR3/Tzb100 cell model of acquired resistance [62]. Studies in animal models have shown that oleuropein administration prevents breast cancer [87], skin cancer [101], and various soft tissue cancers [86]. 

Interestingly, oleuropein showed uroprotective effect against cyclophosphamide-induced hemorrhagic cystitis in a rat model mostly through its antioxidant and anti-inflammatory activities. [102]. Thus, it can be considered as a new anticancer compound for adjuvant therapy not only targeting different stages of cancer development, but also alleviating certain adverse effects in the course of cancer chemotherapy. With respect to the pronounced antioxidant properties of oleuropein, it protects cells from genetic damage, potentially leading to oncogenesis. Anti-angiogenic properties of oleuropein prevent or at least slow down tumor development [86].

## 5. Valuable Properties of Other Plant Polyphenols

Except from the European olive, known as *Olea europea*, there are many other plants rich in invaluable polyphenols. Epigallocatechin and genistein, present in tea and soy, contribute to the DNA hypomethylation, and thus reactivation of the previously suppressed suppressor genes such as *p16*, *RARbeta*, *MGMT*, resulting in the inhibition of cancer induction or growth [103]. Anthocyanins of black raspberries decrease DNA methyltransferase (DNMT1) activity, and also lead to the activation of the suppressor genes via promoter demethylation mechanism [104].

Other kinds of polyphenols, also classified as phytoestrogens, such as resveratrol and genistein, interact with estrogen receptors and influence the regulation of estrogen-dependent gene expression of *P21* gene [105,106].

Resveratrol, as observed in animal models with chemically induced tumor lesions, showed anti-tumor and anti-atherogenic properties. On the other hand, in human tumor cell line models, resveratrol inhibited cell proliferation (cell cycle blockage) with no apoptosis induction [107]. The hydroxytyrosol-rich extracts that are available from different plants induce inhibition of breast cancer cell growth [108]. Interestingly, many studies reported ginger (*Zingiber officinale Roscoe*) to contain numerous chemical constituents of potential health benefits. According to some, pulp and peel of ginger rhizomes exhibit in vitro antiproliferative activity against colon cancer cells. They also show anti-inflammatory properties due to inhibition of cellular nitric oxide production. The anticancer activity of ginger rhizome against colorectal carcinoma is attributed to the main compound of the extract—α-zingiberene and its derivatives [109]. In addition to that, 6-shogaol, another phenolic constituent of ginger, decreased metastatic potential of lung and breast cancer in mice. Studies confirm that 6-shogaol exerts significant anticancer activity both in vivo and in vitro, and at present is concerned as an efficacious immunotherapeutic agent for cancer treatment [110].

Other noteworthy polyphenols are chlorogenic and caffeic acids that are found in coffee and other plants. They are both powerful antioxidants as observed in vitro and they effectively inhibit *N*-nitrosation reaction, as observed in vivo. Furthermore, chlorogenic acid can inhibit DNA damage as observed in vitro since it inhibits lipid peroxidation and suppresses reactive oxygen species-mediated nuclear factor NF-κB [111,112,113]. Interestingly, recent studies have demonstrated a reciprocal relationship between coffee intake and the risk of colon, liver, breast, or endometrial cancer. It was found that the consumption of at least one cup of coffee per day lowered the risk of upper gastrointestinal cancer in the Japanese population by 49% [111,114]. There are, without doubt, plants containing numerous chemicals invaluable for human health that are still waiting to be discovered. 

## 6. Conclusions

**Figure 3:**
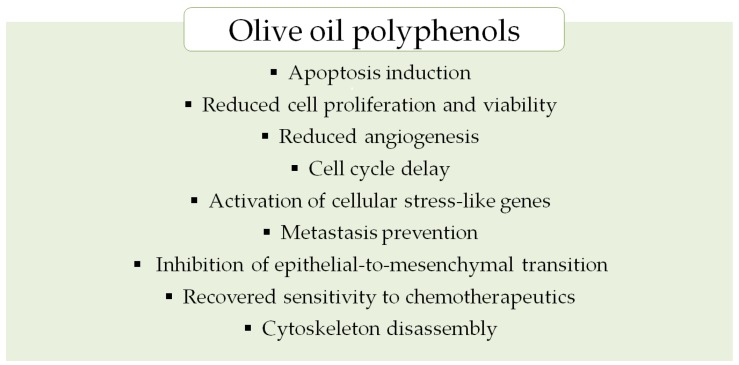
Anticancer effects of olive oil polyphenols [115].

Beneficial roles of natural plant polyphenols in the human body have been known for ages. Their sources are fresh fruits, vegetables and certain processed plant foods. Polyphenols have been reported to reduce morbidity and slow down the progression of cardiovascular, neurodegenerative and cancer diseases. Figure 3 presents a wide range of different benefits of olive oil polyphenols.

The action mechanism of polyphenols is pleiotropic, however, it mostly relates to their antioxidant activity. Natural polyphenols decrease the level of reactive oxygen species protecting biomolecules against oxidative damage. They are also found to modulate the human immune system, affecting proliferation of the white blood cells and the production of cytokines.

Oleuropein, hydroxytyrosol, and their derivatives are polyphenolic compounds that are abundant in olive oil. They are powerful antioxidants displaying anticancer, anti-angiogenic and anti-inflammatory properties.

It should be emphasized that the unique properties of olive oil polyphenols have been evaluated mostly based on in vitro models. In order to verify their beneficial impact on human health, more in vivo studies and well-designed clinical trials are still necessary. Nonetheless, the preliminary results seem to be pretty encouraging in terms of prevention and treatment of cancer or cardiovascular and neurodegenerative diseases.

An increase in consumption of virgin olive oil and other plant products rich in polyphenolic compounds, specifically in populations with low olive oil intake, does seem to be rational and provide diverse health benefits.
